# Supplementary figures and images for: Lactobacillus Mucosae Strain Promoted by a High-Fiber Diet in Genetic Obese Child Alleviates Lipid Metabolism and Modifies Gut Microbiota in ApoE-/- Mice on a Western Diet
Source: Microorganisms. 2020 Aug 12;8(8):1225. doi: 10.3390/microorganisms8081225 (PMC7464838; doi:10.3390/microorganisms8081225)

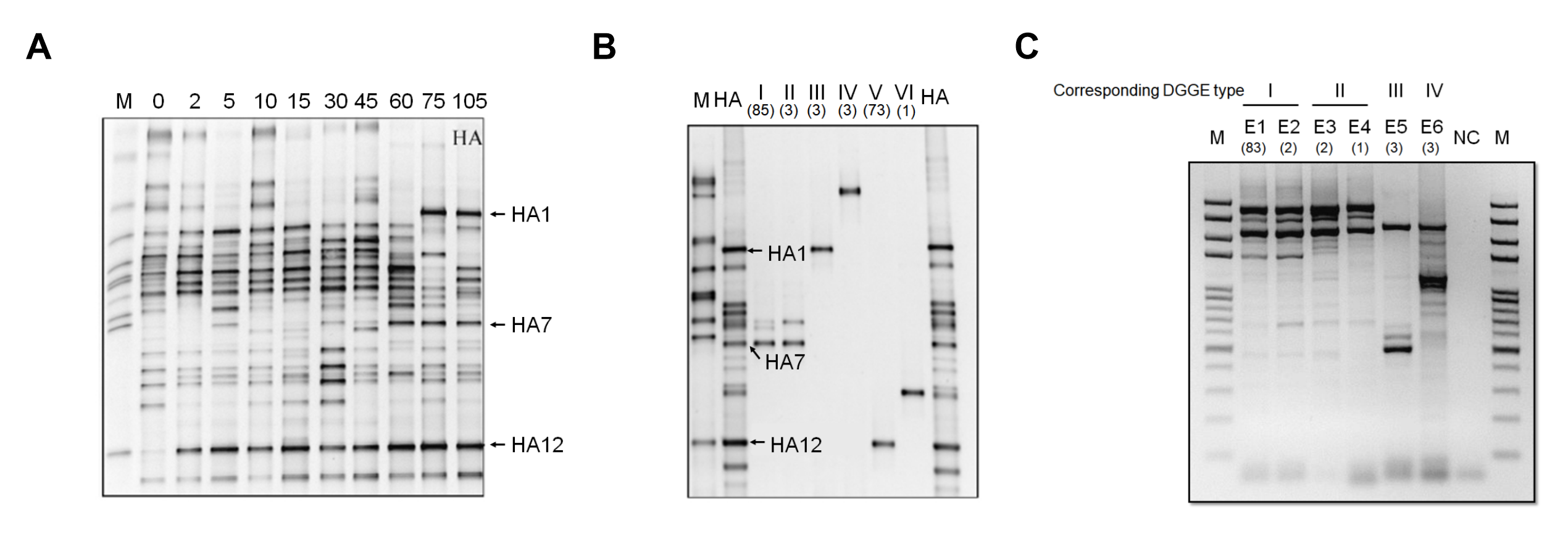

Supplement: Supplementary file 1 [file microorganisms-08-01225-s001.zip › Figure S1.tif]

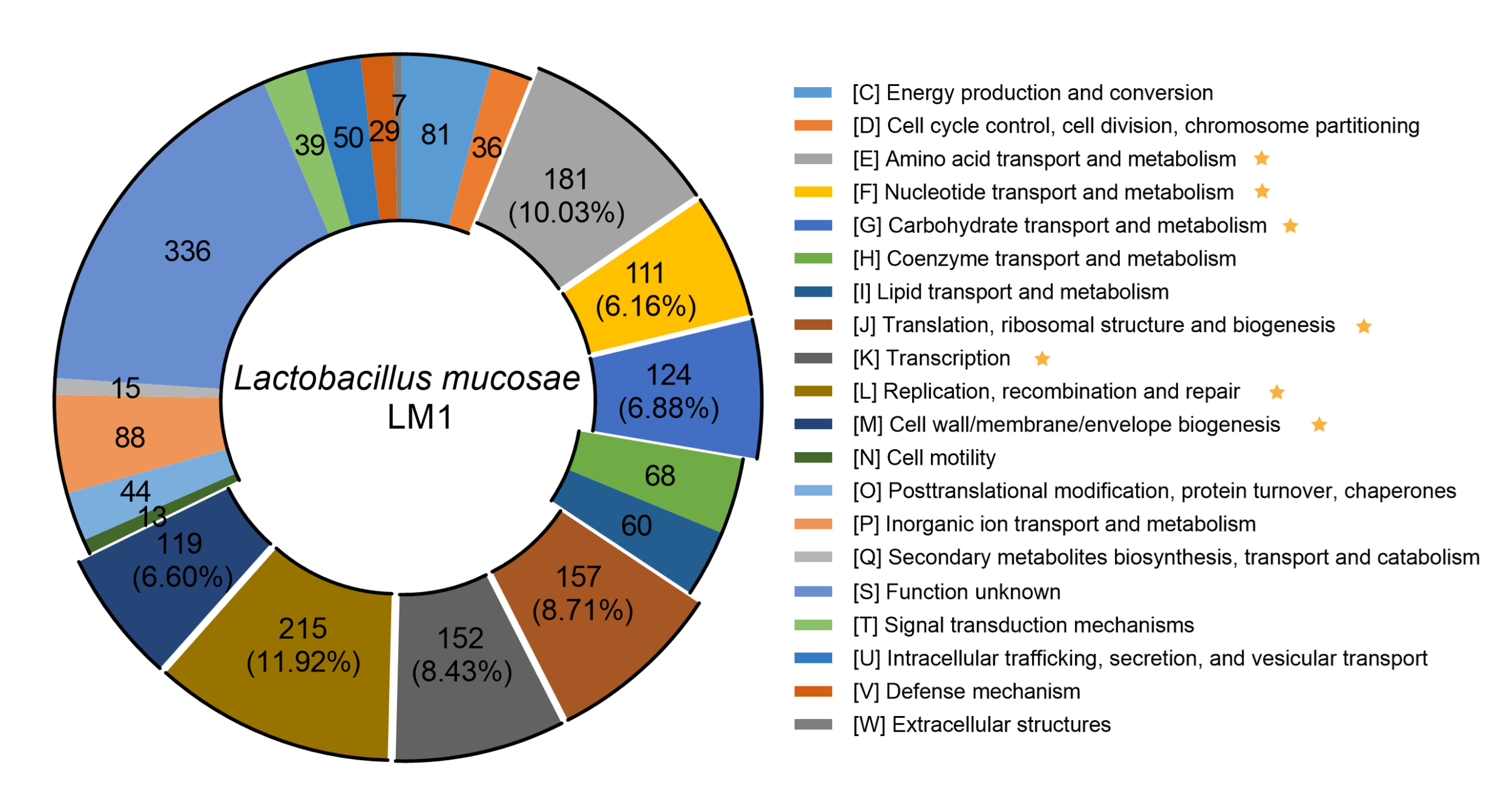

Supplement: Supplementary file 1 [file microorganisms-08-01225-s001.zip › Figure S2.tif]

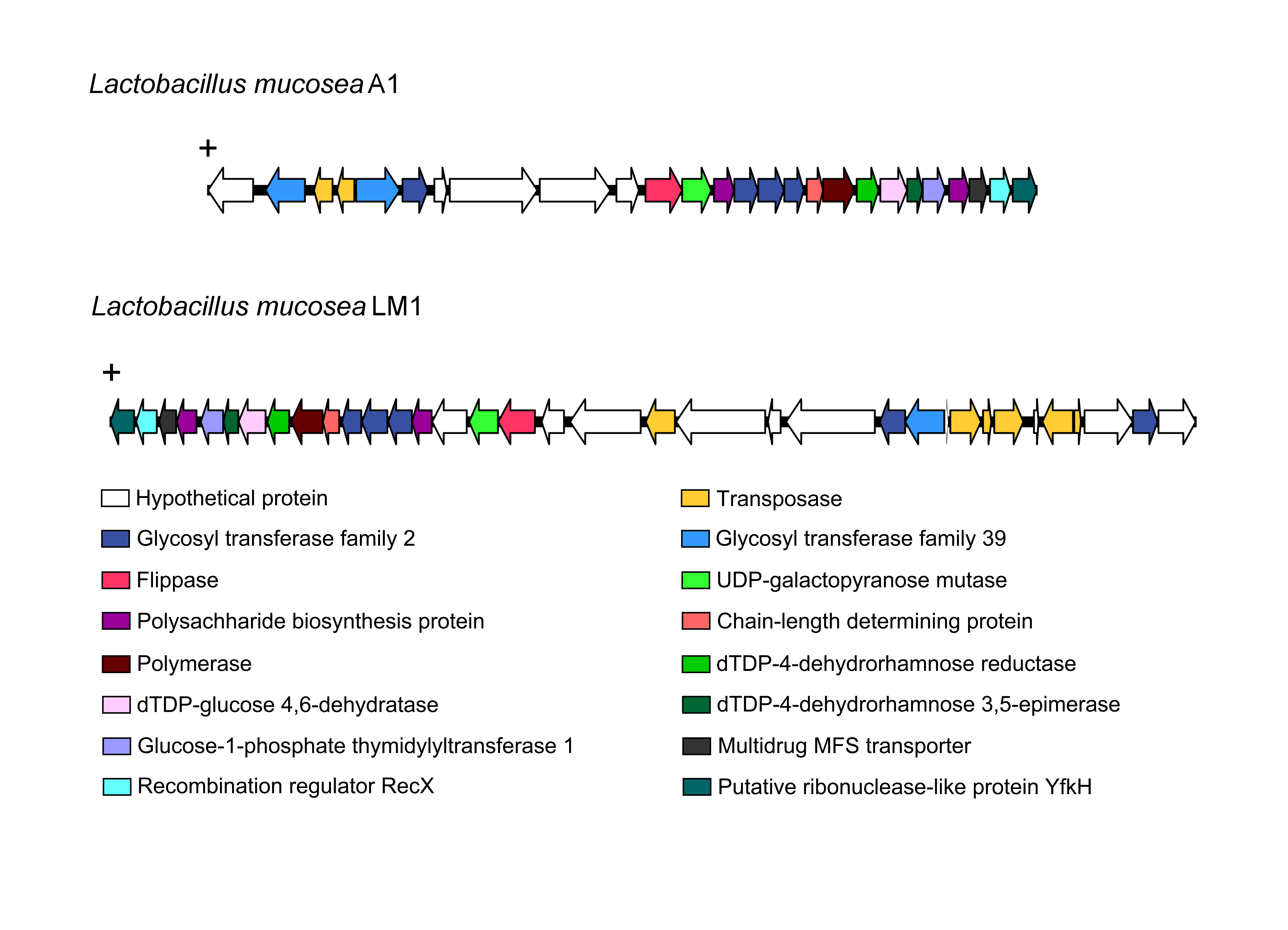

Supplement: Supplementary file 1 [file microorganisms-08-01225-s001.zip › Figure S3.tif]

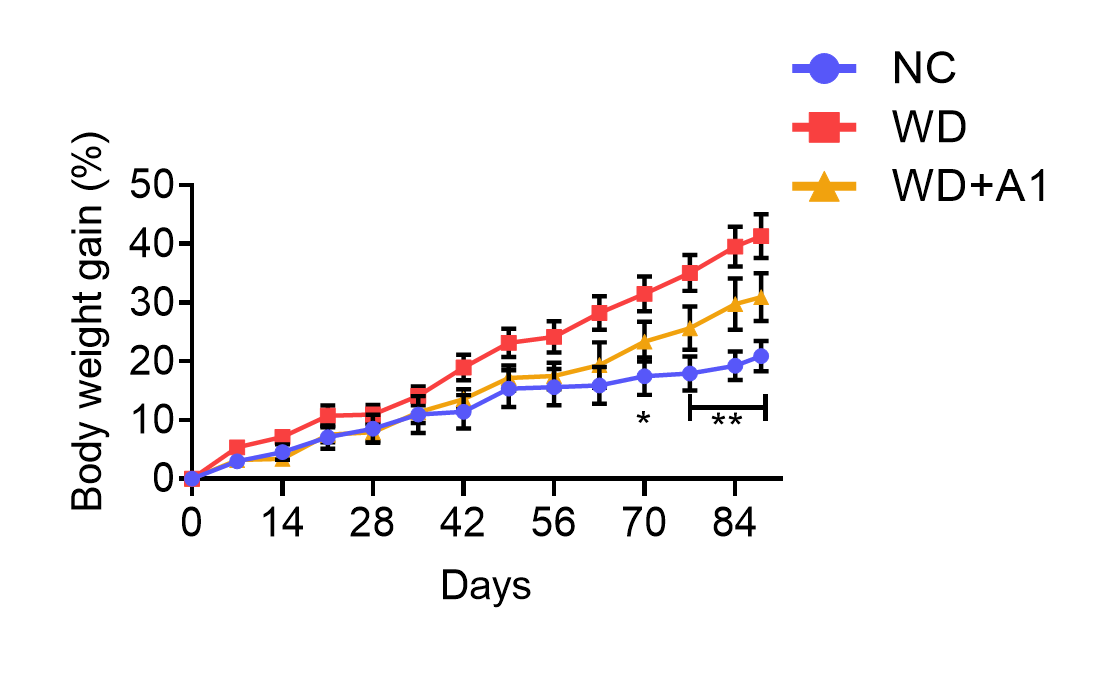

Supplement: Supplementary file 1 [file microorganisms-08-01225-s001.zip › Figure S4.tif]

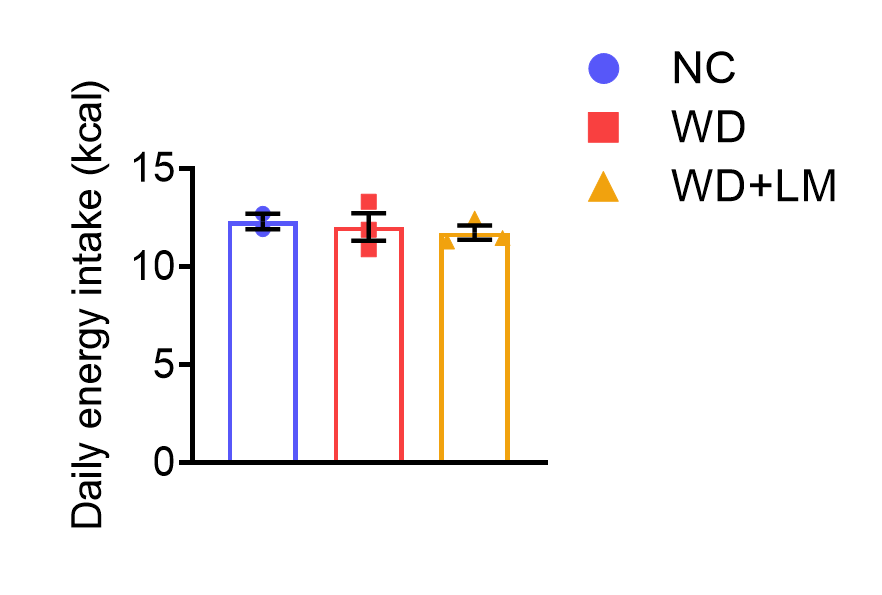

Supplement: Supplementary file 1 [file microorganisms-08-01225-s001.zip › Figure S5.tif]

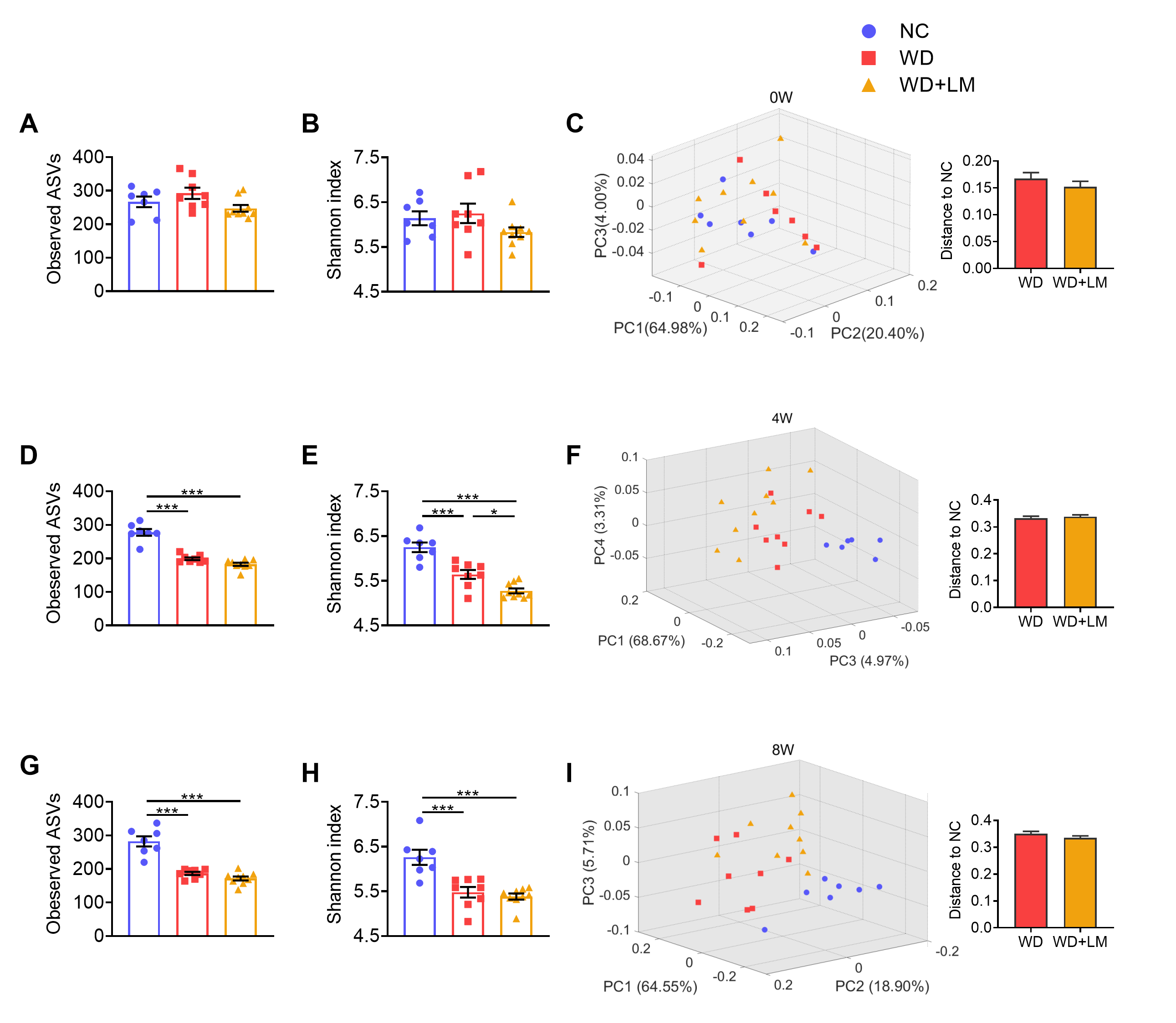

Supplement: Supplementary file 1 [file microorganisms-08-01225-s001.zip › Figure S6.tif]

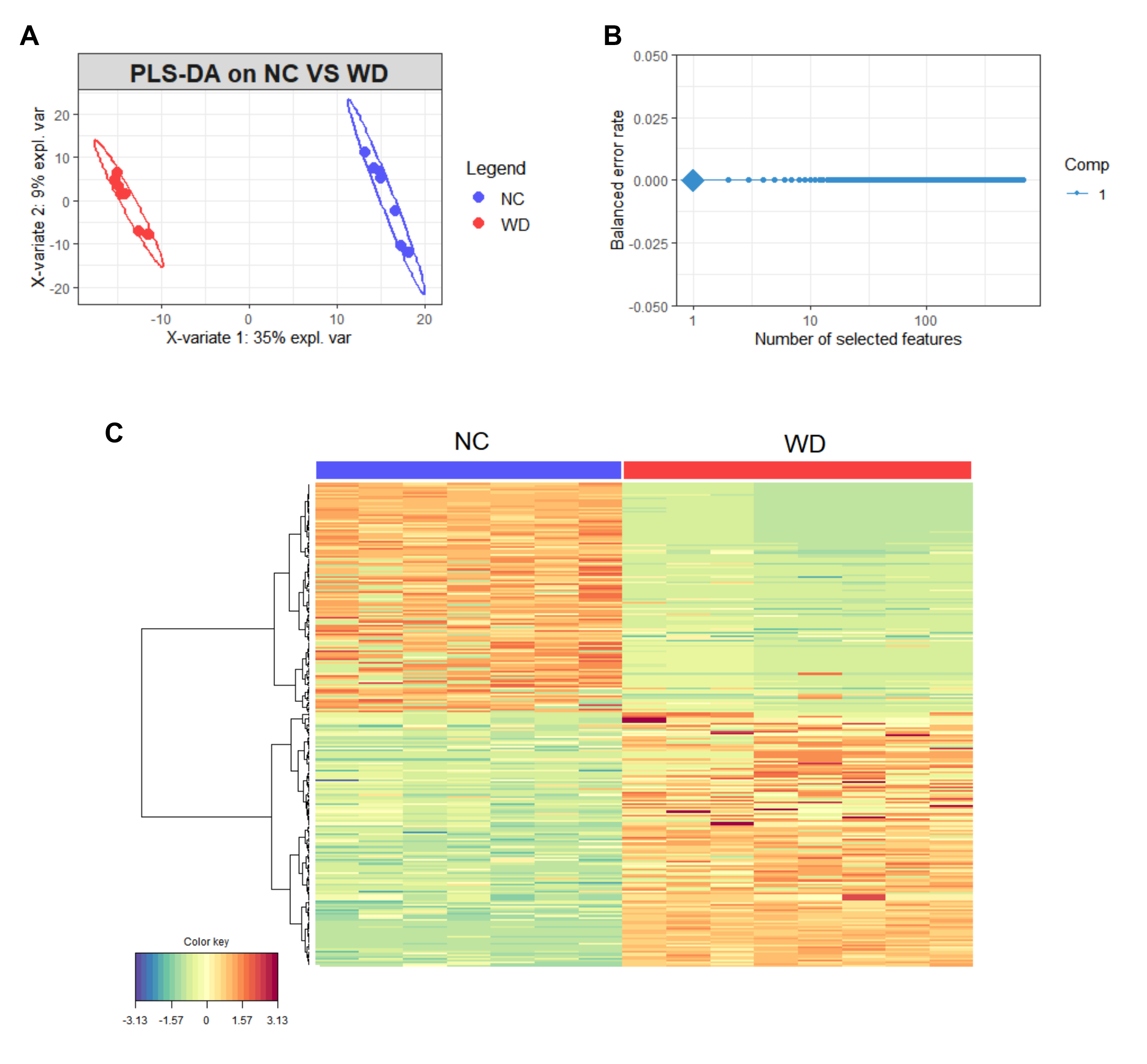

Supplement: Supplementary file 1 [file microorganisms-08-01225-s001.zip › Figure S7.tif]

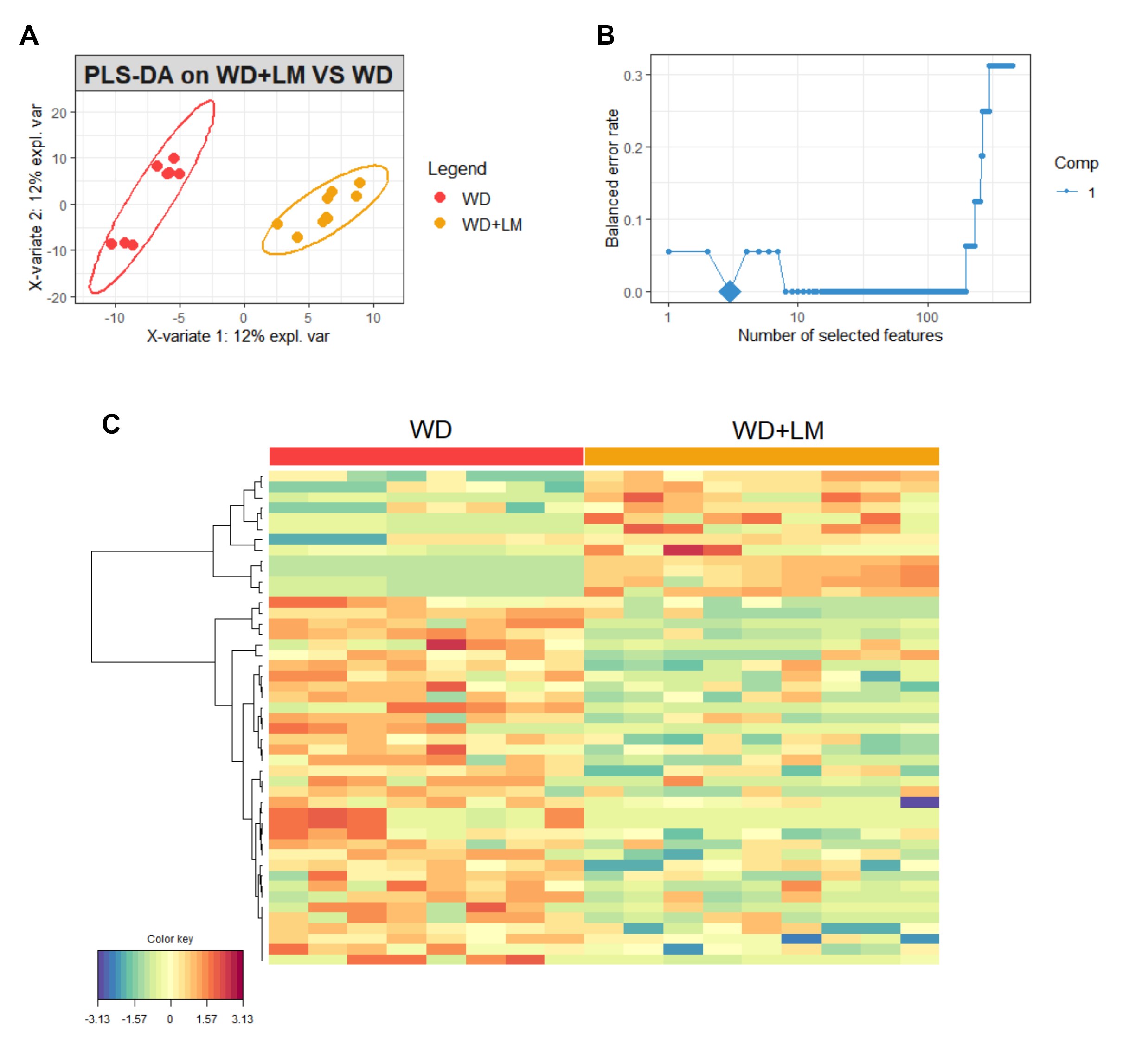

Supplement: Supplementary file 1 [file microorganisms-08-01225-s001.zip › Figure S8.tif]
