# Supplementary material for: Lactobacillus Mucosae Strain Promoted by a High-Fiber Diet in Genetic Obese Child Alleviates Lipid Metabolism and Modifies Gut Microbiota in ApoE-/- Mice on a Western Diet
Source: Microorganisms. 2020 Aug 12;8(8):1225. doi: 10.3390/microorganisms8081225 (PMC7464838; doi:10.3390/microorganisms8081225)
Supplement: Supplementary file 1 [file microorganisms-08-01225-s001.zip › Supplementary Files.doc]

**Supplementary Materials:**

**Figure S1** Sequence-guided isolation of the predominant *Lactobacillus* sp. in the gut of the obese child after 105-days dietary intervention. (**A**) 16S rRNA gene V3 region PCR-DGGE profile of the obese child's fecal samples from 0 day to 105 day during dietary intervention. 0/2/5/10/15/30/45/60/75/105: Fecal sample collected at the 0/2/5/10/15/30/45/60/75/105 days during the intervention. HA1, HA7 and HA12 were bands that were enriched by 105-days intervention and shared 100% sequence similarity with the V3 regions in 16S rRNA gene of *Lactobacillus acidophilus* 30SC, *Lactobacillus mucosae* S32 and *Bifidobacterium pseudocatenulatum* B1279, respectively. (**B**) 16S rRNA V3 region PCR-DGGE profile of 168 isolates and the 105-days fecal sample. 168 isolates were classified into 6 DGGE types, that is I to VI. The numbers in the brackets under the symbols of I to VI mean the numbers of isolates corresponding to different DGGE types. M: DGGE Ladder. HA lane corresponds to the fecal sample collected after 105-days dietary intervention. (**C**) ERIC-PCR banding pattern of 94 *Lactobacillus* isolates from the 105-days fecal sample. The 94 isolates were classified into 6 ERIC types, that is E1 to E6. E1 and E2 belong to DGGE type I, E3 and E4 belong to DGGE type II, E5 belong to DGGE type III and E6 belong to DGGE type IV. The numbers in the brackets under the symbols of E1 to E6 mean the numbers of isolates corresponding to different ERIC types. M: 100 bp Ladder. NC: Negative control.

**Figure S2** Functional categories in the clusters of orthologous groups (COG) analysis of *L.mucosae* LM1. The numbers out the brackets on the circle represents the numbers of genes in each COG category. The numbers in the brackets were the percentages of genes in the corresponding categories to the total genes identified by COG database.

**Figure S3** EPS gene cluster identified in the genomes of *L.mucosae* A1 and *L.mucosae* LM1. Different types of genes were marked with different colors and the products of each type of genes were shown.

**Figure S4** The curve of body weight gain during the whole period of animal trail. Body weight gain was calculated as increased body weight as the percentage of baseline weight for each mouse. All the data are shown are means ± s.e.m. Number of mice per group: NC: 7, WD: 8, WD+LM: 9. Values of each group were analyzed by one-way analysis of variance (ANOVA) followed by Tukey’s *post-hoc* test. **P* < 0.05, ***P* < 0.01: NC VS WD.

**Figure S5** Energy intake of each group. Data are shown as mean ± s.e.m. Number of measure data per group: NC: 2, WD: 3, WD+LM: 3. Values of each group were analyzed by one-way analysis of variance (ANOVA) followed by Tukey’s *post-hoc* test.

**Figure S6** Structure of gut microbiota in ApoE^-/-^ mice at the baseline (0^th^ week) and modulation of gut microbiota after administration of a Western diet with or without *L.mucosae* A1 for 4 weeks and 8 weeks. (**A**, **D**, **G**) ASV richness of gut microbiota at the 0^th^ week (**A**), 4^th^ week (**D**), 8^th^ week (**G**). (**B**, **E**, **H**) Shannon index of gut microbiota at the 0^th^ week (**B**), 4^th^ week (**E**), 8^th^ week (**H**). (**C**, **F**, **I**) PCoA plot of gut microbiota based on Weighted Unifrac distance and Weighted Unifrac distance from the WD group and the WD+LM group to the NC group at the 0^th^ week (**C**), 4^th^ week (**F**), 8^th^ week (**I**). Data for ASV richness, Shannon index and distance are shown as means ± s.e.m. Number of mice per group: NC: 7, WD: 8, WD+LM: 9. Values for ASV richness and Shannon index of each group were analyzed by one-way analysis of variance (ANOVA) followed by Tukey’s *post-hoc* test. Values for distance were analyzed by unpaired *t*-test. **P* < 0.05, ***P* < 0.01, ****P* < 0.001.

**Figure S7** 260 ASVs collected by sPLS-DA model to differentiate gut microbiota structure of ApoE^-/-^ mice in the NC group from the WD group (13^th^ week). (**A**) PLS-DA plot of gut microbiota of mice in the NC and WD groups. (**B**) Balanced error rate of the sPLS-DA model. (**C**) Heatmap of the 260 collected ASVs in the NC and WD groups. The color of spots corresponds to the normalized and log_2_-transformed relative abundance in each sample. The ASVs are clustered by ward.D method.

**Figure S8** 47 ASVs collected by sPLS-DA model to differentiate gut microbiota structure of ApoE^-/-^ mice in the WD+LM group from the WD group (13^th^ week). (**A**) PLS-DA plot of gut microbiota of mice in the WD and WD+LM groups. (**B**) Balanced error rate of the sPLS-DA model. (**C**) Heatmap of the 47 collected ASVs in the WD and WD+LM groups. The color of spots corresponds to the normalized and log_2_-transformed relative abundance in each sample. The ASVs are clustered by ward.D method.

**Table S1** Four contigs composed of the genome of *L.mucosae* A1.

| **Contig** | **Length (bp)** | **GC content (%)** | **Topology** |
| --- | --- | --- | --- |
| **Contig1** | 2,049,443 | 46.83 | circular |
| **Contig2** | 43,519 | 44.27 | circular |
| **Contig3** | 43,513 | 44.27 | circular |
| **Contig4** | 39,346 | 43.84 | circular |

**Table S2** The genomic features of *L.mucosae* A1.

| **Feature** |  | ***L.mucosae* A1** |
| --- | --- | --- |
| **Genome size (bp)** |  | 2,175,821 |
| **G+C content (%)** |  | 46.67% |
| **Number of tRNA genes** |  | 93 |
| **Number of 5S rRNA genes** |  | 8 |
| **Number of 16S rRNA genes** |  | 8 |
| **Number of 23S rRNA genes** |  | 8 |
| **Number of Coding Sequences** |  | 2100 |
| **Protein-coding genes with assigned COG** |  | 1775 (84.52%) |

**Table S3** Genes related to carbohydrate metabolism in the genome of *L.mucosae* A1 and *L.mucosae* LM1 identified by dbCAN database.

| **Enzyme classes and Associated modules** | | **Gene count** | |
| --- | --- | --- | --- |
|  |  | A1 | LM1 |
| **Enzyme Classes** | Glycoside Hydrolases (GHs) | 27 | 22 |
|  | Glycosyl Transferase (GTs) | 24 | 27 |
|  | Carbohydrate Esterase (Ces) | 6 | 6 |
| **Associated Modules** | Carbohydrate-Binding Module (CBMs) | 3 | 2 |

**Table S4** *Q*-values for permutational multivariate analysis of variance (PERMANOVA; 9,999 permutations) based on Weighted Unifrac distance comparing gut microbiota structure of different groups at each time point.

| ***Q*-value** | **NC VS WD** | **NC VS WD+LM** | **WD VS WD+LM** |
| --- | --- | --- | --- |
| **0W** | 0.4856 | 0.2184 | 0.0708 |
| **4W** | 0.00045 | 0.00045 | 0.0859 |
| **8W** | 0.0003 | 0.0006 | 0.0176 |
| **13W** | 0.00015 | 0.00015 | 0.0508 |

**Table S5** The taxonomy and relative abundance of the 47 ASVs altered by *L.mucosae* A1, and the *P* value of Mann-Whitney test evaluating the abundance difference of these ASVs in the NC or WD+LM group compared to the WD group.

| ASV ID | **Taxonomy** | | | Relative abundance (mean ± s.e.m) | | | *P* - value | |
| --- | --- | --- | --- | --- | --- | --- | --- | --- |
|  | Order | Family | Genus | NC | WD | WD+LM | NC VS WD | WD+LM VS WD |
| ASV134 | Clostridiales | Lachnospiraceae | *Lachnoclostridium* | 15.14±6.26 | 3.89±1.89 | 24.44±3.98 | 0.3016 | 0.0005 |
| ASV383 | Clostridiales | Clostridiaceae | *Clostridium* | 0.00±0.00 | 0.00±0.00 | 23.00±9.20 | >0.9999 | 0.0294 |
| ASV176 | Clostridiales | Clostridiaceae | *Clostridium* | 0.00±0.00 | 0.00±0.00 | 84.22±20.07 | >0.9999 | 0.0004 |
| ASV116 | Lactobacillales | Lactobacillaceae | *Lactobacillus* | 0.00±0.00 | 0.00±0.00 | 112.44±34.63 | >0.9999 | 0.0004 |
| ASV018 | Lactobacillales | Lactobacillaceae | *Lactobacillus* | 0.00±0.00 | 0.00±0.00 | 922.89±250.60 | >0.9999 | <0.0001 |
| ASV096 | Lactobacillales | Lactobacillaceae | *Lactobacillus* | 0.00±0.00 | 0.00±0.00 | 147.56±39.73 | >0.9999 | <0.0001 |
| ASV023 | Clostridiales | Ruminococcaceae |  | 32.29±13.54 | 47.38±22.99 | 355.33±120.67 | 0.9176 | 0.0002 |
| ASV159 | Clostridiales | Lachnospiraceae |  | 0.00±0.00 | 12.63±6.13 | 32.11±6.81 | 0.0769 | 0.0537 |
| ASV031 | Clostridiales | Lachnospiraceae | *Acetatifactor* | 3.71±1.49 | 261.63±82.61 | 345.22±45.92 | 0.1803 | 0.5572 |
| ASV624 | Clostridiales | Lachnospiraceae |  | 0.00±0.00 | 0.00±0.00 | 4.22±2.47 | >0.9999 | 0.2059 |
| ASV118 | Clostridiales | Lachnospiraceae |  | 4.71±4.71 | 0.00±0.00 | 43.89±18.44 | 0.4667 | 0.009 |
| ASV393 | Clostridiales | Ruminococcaceae | *Ruminiclostridium* | 3.28±1.32 | 0.00±0.00 | 20.22±9.08 | 0.0256 | 0.0294 |
| ASV027 | Erysipelotrichales | Erysipelotrichaceae | *Turicibacter* | 248.57±55.20 | 174.13±42.36 | 35.67±10.60 | 0.3357 | 0.006 |
| ASV078 | Lactobacillales | Lactobacillaceae | *Lactobacillus* | 55.00±9.76 | 21.00±6.14 | 4.00±2.69 | 0.0082 | 0.029 |
| ASV075 | Clostridiales | Lachnospiraceae |  | 41.86±6.11 | 193.50±52.09 | 26.33±17.89 | 0.0012 | 0.0018 |
| ASV270 | Clostridiales | Ruminococcaceae | *Eubacterium* | 9.14±2.38 | 16.50±10.41 | 0.89±0.89 | 0.4073 | 0.0294 |
| ASV236 | Clostridiales | Lachnospiraceae |  | 24.71±5.83 | 6.38±2.11 | 0.00±0.00 | 0.0034 | 0.009 |
| ASV160 | Clostridiales | Lachnospiraceae |  | 27.57±6.09 | 5.75±2.46 | 0.44±0.44 | 0.0213 | 0.0158 |
| ASV102 | Clostridiales | Lachnospiraceae |  | 65.29±24.89 | 20.13±6.05 | 4.78±4.78 | 0.0766 | 0.0905 |
| ASV219 | Clostridiales | Clostridiales Family XIII | *Eubacterium* | 5.29±1.58 | 30.00±10.56 | 4.00±1.24 | 0.0264 | 0.0036 |
| ASV163 | Clostridiales | Lachnospiraceae | *Acetatifactor* | 0.00±0.00 | 246.25±111.74 | 0.00±0.00 | 0.0769 | 0.0294 |
| ASV282 | Clostridiales | Lachnospiraceae | *Acetatifactor* | 0.00±0.00 | 85.75±38.10 | 0.00±0.00 | 0.0769 | 0.0294 |
| ASV495 | Clostridiales |  |  | 0.00±0.00 | 5.38±1.35 | 0.78±0.57 | 0.0014 | 0.0034 |
| ASV466 | Clostridiales | Ruminococcaceae | *Oscillibacter* | 0.00±0.00 | 10.63±2.44 | 2.56±1.32 | 0.0014 | 0.0081 |
| ASV316 | Clostridiales | Ruminococcaceae | *Ruminiclostridium* | 0.00±0.00 | 49.13±10.64 | 15.33±8.91 | 0.0014 | 0.0298 |
| ASV403 | Clostridiales | Lachnospiraceae |  | 0.00±0.00 | 17.88±4.34 | 0.00±0.00 | 0.0014 | 0.0004 |
| ASV539 | Clostridiales | Ruminococcaceae | *Harryflintia* | 0.00±0.00 | 4.50±0.85 | 1.11±0.61 | 0.0014 | 0.0084 |
| ASV223 | Clostridiales | Ruminococcaceae | *Oscillibacter* | 0.00±0.00 | 40.13±11.20 | 13.00±6.64 | 0.0014 | 0.0521 |
| ASV378 | Clostridiales | Ruminococcaceae | *Ruminiclostridium* | 0.00±0.00 | 16.13±3.60 | 1.00±1.00 | 0.0003 | 0.0002 |
| ASV356 | Clostridiales | Lachnospiraceae |  | 0.00±0.00 | 25.50±13.10 | 2.11±1.11 | 0.0003 | 0.0025 |
| ASV202 | Coriobacteriales | Eggerthellaceae | *Enterorhabdus* | 1.86±1.32 | 19.25±2.72 | 6.22±2.51 | 0.0006 | 0.0035 |
| ASV177 | Clostridiales | Ruminococcaceae |  | 14±7.37 | 90.00±15.38 | 43.33±7.23 | 0.0006 | 0.0037 |
| ASV093 | Clostridiales | Ruminococcaceae |  | 37.86±6.46 | 143.75±21.61 | 80.44±13.44 | 0.0003 | 0.0254 |
| ASV320 | Clostridiales | Clostridiales Family XIII | *Anaerovorax* | 2.57±0.97 | 22.50±3.44 | 10.33±1.94 | 0.0003 | 0.0122 |
| ASV187 | Clostridiales | Ruminococcaceae |  | 0.00±0.00 | 92.13±14.42 | 31.67±14.37 | 0.0003 | 0.0074 |
| ASV130 | Clostridiales |  |  | 4.00±1.21 | 51.38±5.85 | 25.00±4.65 | 0.0003 | 0.0027 |
| ASV194 | Clostridiales |  |  | 3.57±0.69 | 52.50±6.36 | 29.33±3.32 | 0.0003 | 0.0009 |
| ASV217 | Clostridiales | Lachnospiraceae |  | 0.00±0.00 | 48.13±8.69 | 22.67±7.47 | 0.0003 | 0.0487 |
| ASV095 | Clostridiales | Ruminococcaceae | *Ruminiclostridium* | 0.00±0.00 | 105.25±16.60 | 59.22±24.03 | 0.0003 | 0.113 |
| ASV485 | Coriobacteriales | Eggerthellaceae | *Enterorhabdus* | 0.71±0.71 | 8.38±2.27 | 2.00±0.93 | 0.0067 | 0.0119 |
| ASV112 | Clostridiales | Lachnospiraceae |  | 13.14±3.02 | 112.88±23.33 | 31.44±7.81 | 0.0003 | 0.0017 |
| ASV113 | Clostridiales | Lachnospiraceae |  | 1.71±1.13 | 72.63±17.40 | 32.22±10.92 | 0.0003 | 0.1292 |
| ASV559 | Clostridiales | Clostridiales Family XIII | *Eubacterium* | 0.00±0.00 | 3.38±1.03 | 0.67±0.67 | 0.007 | 0.0232 |
| ASV215 | Clostridiales | Lachnospiraceae |  | 0.00±0.00 | 129.00±47.24 | 0.00±0.00 | 0.0256 | 0.009 |
| ASV345 | Clostridiales | Lachnospiraceae |  | 0.00±0.00 | 8.00±2.10 | 2.11±1.40 | 0.007 | 0.0448 |
| ASV474 | Erysipelotrichales | Erysipelotrichaceae | *Turicibacter* | 0.00±0.00 | 16.63±6.75 | 0.00±0.00 | 0.0769 | 0.0294 |
| ASV128 | Lactobacillales | Enterococcaceae | *Enterococcus* | 0.29±0.29 | 6.88±1.61 | 2.44±1.25 | 0.0005 | 0.0339 |
